# Pulmonary artery pressure assessed by catheterization and its concordance with transthoracic echocardiographic estimates in patients with pulmonary arterial hypertension: experience of the Colombian Pulmonary Hypertension Network in a real-life study

**DOI:** 10.62675/2965-2774.20250182

**Published:** 2025-05-06

**Authors:** Mauricio Orozco-Levi, Alejandro Londoño, Rafael Conde, Manuel Conrado Pacheco Gallego, Julián Cortes Colorado, Carlos Jaime Velázquez, Ricardo Gómez Palau, Lucila Teresa Flórez de Arco, Juliana De Luque, Ana Maria Pérez-Zauner, Alba Ramírez-Sarmiento

**Affiliations:** 1 Respiratory Department Hospital Internacional de Colombia Floridablanca Colombia Respiratory Department, Hospital Internacional de Colombia – Floridablanca, Colombia.; 2 Respiratory Department Clinica Cardio VID Medellín Colombia Respiratory Department, Clinica Cardio VID - Medellín, Colombia.; 3 Fundación Neumológica Colombiana Bogota Colombia Fundación Neumológica Colombiana - Bogota, Colombia.; 4 Respiratory Care Unit RESPIREMOS Pereira Colombia Respiratory Care Unit, RESPIREMOS - Pereira, Colombia.; 5 Pneumology Department Clinica de Neumología Del Pacífico Cali Colombia Pneumology Department. Clinica de Neumología Del Pacífico - Cali, Colombia.; 6 Rheumatology Department Clinica Bolivariana Medellín Colombia Rheumatology Department, Clinica Bolivariana - Medellín, Colombia.; 7 Cardiology Department Imbanaco Medical Center Cali Colombia Cardiology Department. Imbanaco Medical Center - Cali, Colombia.; 8 Epidemiology Department Medical Writing Bogota Colombia Epidemiology Department, Medical Writing - Bogota, Colombia.; 9 Hospital Internacional de Colombia Floridablanca Colombia Research Unit, EMICON Group, Hospital Internacional de Colombia – Floridablanca, Colombia.

**Keywords:** Pulmonary arterial hypertension, Pulmonary artery, Blood pressure, Cardiac catheterization, Echocardiography

## Abstract

**Objective:**

To evaluate the correlation and concordance of pulmonary artery systolic pressure values measured via right heart catheterization and estimated via transthoracic echocardiography based on data from a multicenter cohort of patients with pulmonary hypertension in Colombia.

**Methods:**

A retrospective study was conducted of patients with pulmonary hypertension classified into Groups 1 or 4 according to the definitions of the ESC/ERS-PH-2022 guidelines. Patients were obtained from the Colombian Pulmonary Hypertension Network (HAPredco) database.

**Results:**

A total of 633 patients were identified and included in this study. Among these patients, 77.7% (n = 492) had complete data from transthoracic echocardiography at diagnosis, 58,3% (n = 369) had complete data from right heart catheterization at the time of diagnosis, and 264 (41.7%) had complete data from both tests at diagnosis, with a difference in days between them of 1 (84). The values of pulmonary artery systolic pressure estimated by transthoracic echocardiography and those obtained by right heart catheterization were significantly correlated (p < 0.001) in the entire population evaluated, as was the correlation assessed for those patients with a gap of ≤ 7 days (p = 0.0001) or ≤ 48 hours (p = 0.041) between the two examinations; however, these findings presented a low Spearman (0.32 for ≤ 7 days and 0.264 for ≤ 48 hours) and Lin´s correlation coefficient (0.32 for ≤ 7 days and 0.21 for ≤ 48 hours).

**Conclusion:**

The pulmonary artery systolic pressure values estimated via transthoracic echocardiography and measured via right heart catheterization were significantly but weakly linearly correlated, with low concordance. These findings suggest interindividual variability between the pulmonary artery systolic pressure values obtained by the two methods, which may have clinical significance in follow-up and decision-making.

## INTRODUCTION

Pulmonary hypertension (PH) is a hemodynamic syndrome defined as an increase in pulmonary pressure due to intrinsic alterations of the pulmonary vasculature or secondary to cardiac, pulmonary, infectious, or other pathologies.^(1)^ The classification of patients with PH is based on their etiology, which allows their grouping into five sets of different clinical entities that may have variations in the hemodynamic threshold, with specific (pathological and hemodynamic) findings and treatment strategies for those with an underlying disease.

**Figure 1:**
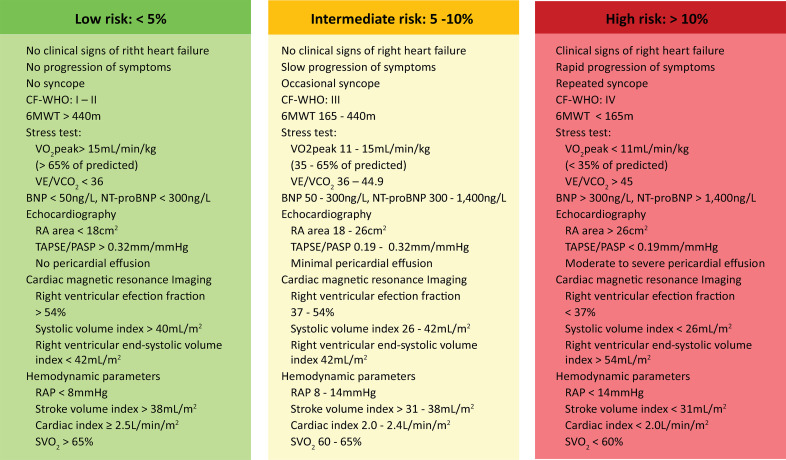
Risk assessment defines 1-year mortality in pulmonary arterial hypertension patients. CF-WHO - World Health Organization functional class; 6MWT - 6-minute walk test; VO_2_ - oxygen consumption; VE/VCO_2_ - ratio of minute ventilation to carbon dioxide production; BNP - brain natriuretic peptide; NT-proBNP: N-terminal pro-brain natriuretic peptide; RA - right atrium; TAPSE - tricuspid annular plane systolic excursion; PASP - pulmonary arterial pressure; RAP - right atrial pressure; SVO_2_ - mixed venous blood oxygen saturation. Risk was defined as 1-year mortality.

Source: Humbert M, Kovacs G, Hoeper MM, Badagliacca R, Berger RM, Brida M, et al.; ESC/ERS Scientific Document Group. 2022 ESC/ERS Guidelines for the diagnosis and treatment of pulmonary hypertension. Eur Heart J. 2022;43(38):3618-731.^(13)^

Pulmonary arterial hypertension (PAH) corresponds to a syndrome that derives from the restriction of pulmonary artery flow and is defined as a mean pulmonary artery pressure (mPAP) > 20mmHg and pulmonary vascular resistance (PVR) > 3 Wood units (uW) at rest.^(1,2)^It is a progressive vasculopathy secondary to pathological remodeling of small pulmonary vessels, which can lead to right heart failure and death, and it is associated with a negative impact on quality of life.^(1)^Observational studies, followed by clinical guidelines, have allowed the identification of predictive prognostic arrays (derived from clinical, paraclinical, demographic, and imaging variables),^(3-6)^through simple probabilistic^(3,7-11)^ or multiparametric^(12-14)^ morbidity and mortality risk models. These risk stratifications make it possible to suggest treatment choices and influence patient follow-up and referral to reference centers. However, these predictive risk scales are derived from the interaction of multiple variables. To date, there is no rigorous external validation process to determine the weight of each variable in the probabilistic model or the minimum number of variables that allow adequate risk inference in Colombia. Recently, Díez et al. (Argentina) validated the use of three noninvasive variables (functional class according to the World Health Organization, the 6-minute walk test (6MWT), and brain natriuretic peptide or N-terminal pro-brain natriuretic peptide fraction) for risk assessment in patients with PAH.^(12)^ Meanwhile, the European Society of Cardiology (ESC)/European Respiratory Society (ERS) 2022 management guidelines^(13)^ propose risk stratification by 13 prognostic variables (Figure 1), and data from the US registry (REVEAL) use a total of 12 variables to stratify risk.^(13,15)^

The ability of the right ventricle to adapt to this overload plays a fundamental role in symptoms, survival, and death, since right heart failure is the leading cause of death in these patients.^(4,16)^Under this premise, assessing cardiac function by determining hemodynamic patterns in patients with PAH is highly important; this can be assessed by right heart catheterization (RHC), with mean right atrial pressure and cardiac output being predictors of survival in patients with PAH.^(3,5,11,17)^In addition, previously described factors, such as transthoracic echocardiography (TTE), the 6MWT, pulmonary function tests, and serological markers, are systematically measured and used in risk stratification, allowing the comprehensive assessment of PAH patients.

Transthoracic echocardiography is a valuable method for estimating ventricular function and systolic pulmonary arterial pressure (PASP). Echocardiography calculates pulmonary systolic pressure based on Bernoulli’s principle (∆P = 4 x V^2^), where the maximum velocity of the tricuspid regurgitation jet can be used to estimate right ventricular systolic pressure by adding the right atrial pressure. Its usefulness is based on its low cost and the possibility of standardizing the measurement technique. However, intraobserver and interobserver variability and the mathematical basis for estimating pressure from a regurgitation jet at the tricuspid valve make its correlation with direct pressure measurement significant but weak. This imperfect correlation may lead to incorrect decisions when worsening is underestimated or when improvement is assumed as a response to a specific therapy.

Several studies have evaluated the relationship between noninvasive and invasive methods for assessing pulmonary artery pressure. A recent meta-analysis compared transesophageal echocardiography (TEE) with RHC across 27 different studies and reported a combined sensitivity of 0.78 and specificity of 0.68 for PASP and a sensitivity of 0.88 and specificity of 0.62 for right ventricular systolic pressure (sRVP).^(18)^The same study reported an overall sensitivity of 0.82 and specificity of 0.71 for detecting PH. In a study from the United States, a single reference center compared the reliability of noninvasive PAP assessment by TTE with that of invasive measurements in a population of 307 patients, revealing a strong correlation (r = 0.89) compared with both a derivation cohort and a validation sample.^(19)^This result was consistent with findings from a more extensive multicenter study involving 1,695 patients, which also demonstrated a strong correlation.^(20)^

Nevertheless, a significant correlation does not necessarily translate into an identity of values. Systolic pulmonary arterial pressure measured with RHC and TTE is often considered very similar or equivalent in Colombia, an assumption that may have implications for initial diagnostic interpretation or patient follow-up; this has motivated the present study to analyze data from a multicenter cohort of patients with PAH, evaluating the correlation and concordance between PASP values measured by RHC and those estimated by TTE in patients diagnosed with PAH or chronic thromboembolic pulmonary hypertension (CTEPH) in Colombia.

## METHODS

### Study design

Approval was obtained from the ethics committee of each institution. A retrospective cohort study was performed to analyze the data of all patients diagnosed with pulmonary hypertension corresponding to Groups 1 or 4, according to the ESC/ERS-PH-2022 guidelines. The source of information is a multicenter registry of nine national hospitals consolidated through the Colombian Pulmonary Hypertension Network database. This database includes incident and prevalent patients with records from 1986 to 2022. Sociodemographic (age, sex, marital status, occupation, and origin), clinical (weight, height, body mass index, presence of dyspnea, syncope, and chest pain cause of PH, associated comorbidities), imaging (RHC and TTE) and paraclinical (6MWT, spirometry, atrial natriuretic peptide, arterial blood gases, and liver enzymes) variables of patients at diagnosis were included. All data were anonymized, and the present study was performed under regulations established by the Declaration of Helsinki in its current version.

### Study population

All medical records at the participating institutions were reviewed. Information from adult patients with confirmed PH classified into PH Group 1 (PAH) or PH Group 4 (CTEPH) according to the ESC/ERS-PH-2022 guideline definitions^(13)^were included. Patients belonging to PH Group 1 were defined as those with PAH whose origin was related to heredity (mutations in BMPR2 or other mutations, drugs or toxins, and associated with connective tissue diseases, human immunodeficiency virus infection, portal hypertension, congenital heart disease, or schistosomiasis). Patients in PH Group 4 were defined as those who presented with pulmonary hypertension secondary to chronic thromboembolic disease and other pulmonary artery obstructions. For the comparative analysis of RHC and TTE, only those patients in the registry who had both tests were included, and the time elapsed between the two tests was calculated. The research group reviewed the RHC data to verify the information required to calculate pulmonary vascular resistance. Echocardiography data were recorded regarding the tricuspid regurgitation jet velocity and its surrogate variable of systolic pressure. Variables were included according to the clinical recording protocols of the treating physicians and the availability of these at the time of recording. Sociodemographic variables, descriptive variables of the disease in the initial assessment, initial paraclinical variables, risk analysis, treatments, and outcomes were determined. An Excel (version 17) spreadsheet was constructed for the variables previously described. A 100% data verification was performed as a quality control, and the data tabulator was a third-party external to the research. Patients were evaluated, treated, and followed up according to the treating physician’s criteria under a nonbinding academic standardization of the NIZA 2018 consensus.^(21)^

### Sample size

Accounting for an anticipated 20% loss to follow-up, the final sample size was adjusted to 372 patients. This calculation was based on a disease prevalence of 28/1,000,000 according to the 2018 prevalence reports for Colombia.^(1)^

### Statistical analysis

Statistical analyses were performed via the statistical science package (SPSS version 26) through descriptive and inferential statistics for the entire registry population at the cutoff date. An alpha error of 0.05, a beta error of 80% with a confidence interval of 95% (95%CI), and a statistically significant value of p less than 0.05 were determined. The normality of the samples was determined via the Kolmogorov–Smirnov test. Univariate analysis was performed by summary statistics, charts, and frequency distributions, with the mean±standard deviation or median (interquartile range) reported according to the normality test for numerical variables and frequencies for categorical variables. A bivariate analysis evaluated the associations between patients’ clinical and paraclinical variables and treatment, stratification, functional deterioration, death, hospitalization, and follow-up paraclinical variables, reporting those that were statistically significant. A subanalysis of patients with wedge pressures greater than or equal to 15 mmHg was performed. Chi-square, ANOVA, and Kruskal–Wallis tests were used to evaluate differences. The correlation was assessed in patients with all the variables taken by RHC and estimated by TTE. The data were subsequently grouped according to the time gap between RHC and TTE; two subgroups were created, the first with an interval of ≤ 7 days between one examination and the other and the second with an interval of ≤ 48 hours. Spearman’s Rho correlation coefficient and Lin’s correlation coefficient were calculated for these data. Bland–Altman plots and heat and dendrogram plots were generated for the concordance analysis.

## RESULTS

Information at diagnosis was available for 633 adult patients aged 50 ± 17 years, 71.2% of whom were female. Most of the patients were from urban areas [53.1% (n = 336)] and were affiliated with the contributory health regime [65.7% (n = 416)] (Table 1).

**Table 1:** Sociodemographic characterization of the patients (n = 633)

Variables	n (%)	Mean ± SD
Age (years)		50.48 ± 16.82
Weight (kg)		61.84 ± 15.05
Height (m)		156.71 ± 18.57
BMI (kg/m^2^)		23.61 ± 5.8
BSA (m^2^)		2.35 ± 4.16
Sex		
Male	451 (71.2)	
Female	182 (28.8)	
Payer type		
Contributory regimen	416 (65.7)	
Subsidized regimen	101 (16)	
Health policy	8 (1.3)	
Urban	336 (53.1)	
Rural	61 (9.6)	

SD - standard deviation; BMI - body mass index; BSA - body surface area.

**Figure 2:**
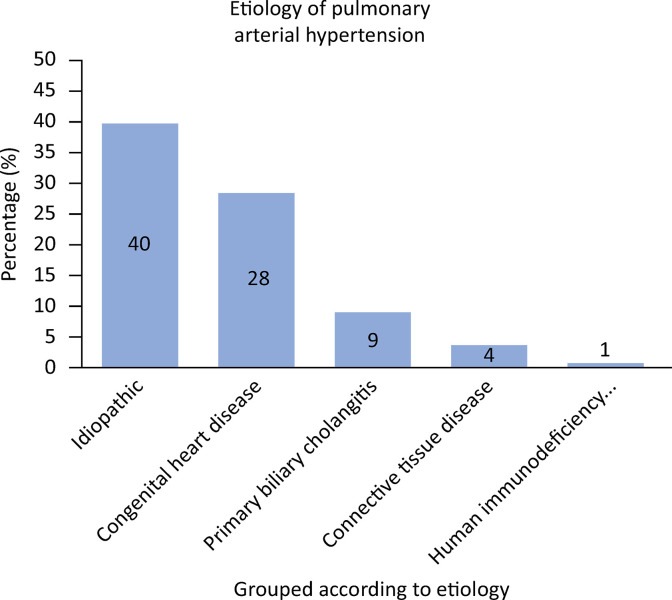
Distribution of patients in Group 1 with pulmonary hypertension.

The frequency for each PH group was as follows: Group 1 [62% (n = 393)], Group 4 [0.2% (n = 1)], and Groups 1 and 4 [37.75% (n = 239)]. The most common cause of heart disease in patients in Group 1 was idiopathic (39%), followed by congenital heart disease (28%) (Figure 2).

**Figure 3:**
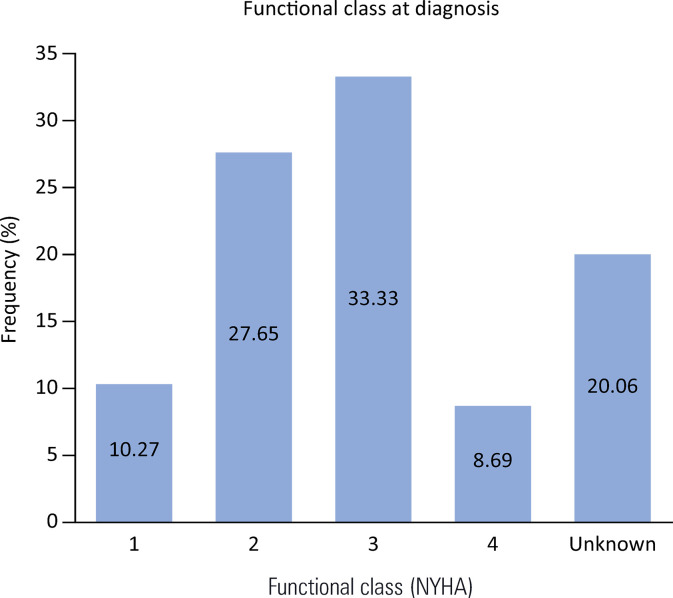
New York Heart Association functional classes. Distribution of percentages of the study patients in each NYHA functional class. NYHA - New York Heart Association.

In the analysis of symptoms recorded at the initial evaluation, 17% (n = 106) of the patients reported dyspnea, 6% (n = 37) reported chest pain, and 3% (n = 21) reported syncope. A total of 33.3% (n = 211) of the patients had a functional class (New York Heart Association [NYHA] Classification) of 3 at that time, 27.65% (n = 177) had a functional class of 2, and 10.27% (n = 65) had a functional class of 1 (Figure 3). At the end of the follow-up, 21.2% (n = 134) of the subjects had died.

For 405 (64%) of the patients, initial RHC data (time of diagnosis) were available at the cutoff time for the present study’s analysis. The mean pulmonary artery pressure was 53 ± 18mmHg with a mean wedge pressure of 14 ± 7.0mmHg (Table 2). Ninety-three patients had a wedge pressure greater than or equal to 15mmHg and were consequently defined as having combined PAH-CTEPH.^(12)^ In these patients, the mean wedge pressure was 22 ± 7mmHg, and the most frequent diagnosis was idiopathic PAH [62.36% (n = 58)], followed by congenital heart disease [24.43% (n = 40)]; the most frequent heart disease was Eisenmenger syndrome [37% (n = 34)]. The other diagnoses corresponded to pulmonary veno-occlusive disease [8.6% (n = 8)] and connective tissue disease [6.45% n = 6)] and were associated with anorexia [2.15% (n = 2)]. A bivariate analysis was performed to determine eventual differences between patients with combined PAH and those with pulmonary wedge pressures less than 15mmHg.

**Table 2:** Diagnostic and paraclinical methods available in the clinical records at the initial evaluation of patients

Variables at rest	Mean ± SD
Right heart catheterization (n = 405)	
PASP (mmHg)	88.49 ± 26.66
PADP (mmHg)	34.95 ± 15.05
PAMP (mmHg)	52.89 ± 17.62
PAPP (mmHg)	57.18 ± 17.50
PAOP (mmHg)	14.42 ± 6.96
LVEDP (mmHg)	10.63 ± 4.69
PVR (Wu)	11.45 ± 7.96
RAP (mmHg)	9.56 ± 6.08
Cardiac output (L/min)	4.38 ± 1.66
Cardiac index (L/min/m^2^)	2.45 ± 1.03
Transpulmonary gradient (mmHg)	41.34 ± 20.57
Transthoracic echocardiography (n = 264)	
PASP (mmHg)	80.17 ± 26.88
LVEF (%)	56.80 ± 9.53
TAPSE (mm)	16.69 ± 6.24
Dilated right cavities (%)	11.37*
Decreased collapse of IVC (%)	2.21*
Pericardial effusion (%)	2.84*
6mWT (n = 322)	
FiO_2_ (%)	24.44 ± 5.05
SpO_2_ (%)	91.26 ± 4.17
SpO2_final_ (%)	81.86 ± 9.68
Walked distance (m)	439.20 ± 120.26
Walked distance (%ref)	58.45 ± 18.99
HR (bpm)	79.56 ± 13.54
HR (%ref)	53.93 ± 9.28
HR_peak_ (bpm)	122.77 ± 23.50
HR_peak_ (%ref)	67.73 ± 12.98
CPET (n = 16)	
FEV_1_ (L)	2,58 ± 0,87
FEV_1_ (%ref)	77.71 ± 12.18
W_peak_ (watt)	72.57 ± 40.78
W_peak_ (%ref)	48.07 ± 19.48
Initial SpO_2_ (%)	92 ± 5
Minimum SpO_2_ (%)	85 ± 8
SpO_2 peak_ (%)	85 (8)
Baseline *versus* peak exercise SpO_2_ (%)	-7 ± 5
VO_2 peak_ (mL/kg/min)	13.29 ± 4.08
VO_2 peak_ (%ref)	52.71 ± 23.86
HR (bpm)	88 ± 10.18
HR (%ref)	50.87 ± 6.74
HR_peak_ (bpm)	141.56 ± 21.79
HR_peak_ (%ref)	81.25 ± 8.87
VO_2peak_/HR_peak_ (mL/kg/min/bpm)	6.79 ± 1.89
VO_2peak_/HR_peak_ (%ref)	56.93 ± 13.36
RQ_peak_	1.14 ± 0.36
RQ_max_ at recovery	1.29 ± 0.47
VE_peak_ (L/min)	53.47 ± 19.90
VE_peak_ (%MVV)	60.09 ± 12.70
MVV (L/min)	107.62 ± 28.93
MVV (%ref)	89.56 ± 18.62
LT (mL/kg/min)	9.83 ± 3.01
LT (% predicted VO_2max_)	37.36 ± 21.25
Systolic BP (mmHg)	113.57 ± 13.47
Diastolic BP (mmHg)	78.07 ± 7.14
Systolic BP_peak_ (mmHg)	146.21 ± 27.24
Diastolic BP_peak_ (mmHg)	93.64 ± 18.14
VO_2peak_/Watt_peak_ (mL/kg/min/W)	15.5 ± 5.73
Arterial blood gases and other laboratory tests (n = 108)	
P_a_O_2 at rest_ F_i_O_2_ = 0.21 (mmHg)	68.68 ± 23.17
P_a_CO_2 at rest_ F_i_O_2_ = 0.21 (mmHg)	34.91 ± 8.99
HCO_3_ (meq/L)	23.00 ± 5.32
BNP (pg/mL)	502 ± 2199
Prothrombin time (sec)	19.37 ± 10.41
Partial thromboplastin time (sec)	41.02 ± 16.20
International normalized ratio	1.60 ± 1.06
Creatinine (mg/dL)	0.96 ± 0.28
AST (mg/dL)	73.51 ± 265.26
ALT (mg/dL)	47.96 ± 119.08
Bilirubin (mg/dL)	0.97 ± 1.26
Direct bilirubin (mg/dL)	0.64 ± 1.54

PASP - pulmonary artery systolic pressure; PADP - pulmonary artery diastolic pressure; PAMP - pulmonary artery mean pressure; PAPP - pulmonary artery pulse pressure; PAOP - pulmonary artery occlusion pressure; LVEDP - left ventricle end-diastolic pressure; PVR - pulmonary vascular resistance; RAP - right atrium pressure; LVEF - left ventricle ejection fraction; TAPSE - tricuspid annular plane systolic excursion; IVC - inferior vena cava; 6mWT - 6 minute walking test; FiO_2_ - inspired faction of oxygen; SpO_2_ - oxyhemoglobin saturation assessed by pulse-oximetry; HR - heart rate; CPET - comprehensive cardiopulmonary exercise test; FEV_1_ - forced expiratory volume; W - watt; VO_2_ - oxygen uptake; RQ - respiratory quotient; VE - minute ventilation; MVV - maximum voluntary ventilation; LT - lactate threshold; BP - blood pressure (noninvasive measurement); PaO_2_ - partial pressure of oxygen; PaCO_2_ - partial pressure of carbon dioxide; HCO_3_ - bicarbonate; BNP - brain natriuretic peptide; AST - aspartate aminotransferase; ALT - alanine transaminase.

* Corresponds to percentages.

**Figure 4:**
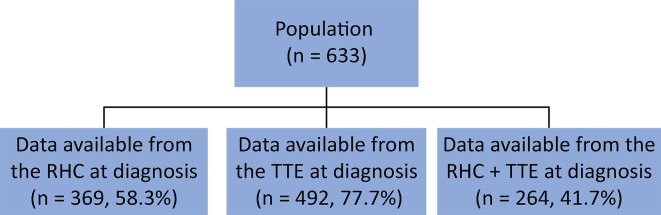
Diagram of diagnostic exams. Diagram of patients with transthoracic echocardiography, right heart catheterization or both. RHC - right heart catheterization; TTE - transthoracic echocardiography.

For 264 (41.7%) of the patients, both RHC and TTE data at diagnosis were available, with a difference in days between them of 1 (84) (Figure 4). On TTE, the estimated PASP was 80.17 ± 26.88mmHg, and the mean tricuspid annular plane systolic excursion (TAPSE) was 16.69 ± 6.24 mm (Table 2).

Three hundred twenty-two patients (50.86%) had an initial 6MWT, with a mean distance of 439.2 ± 20.26 meters (58.45% of the predicted value). Only 16 patients (2.52%) had integrated cardiopulmonary exercise testing information available, and 108 (17%) had resting arterial blood gases (Table 2). The mean brain natriuretic peptide value was 560.92 ± 1318,28pg/mL. As additional diagnostic methods, 10.90% (n = 69) of patients had baseline computer-based lung vessel tomography, and 6.16% (n = 39) had ventilation/perfusion scans. Concerning pulmonary pressure determined during RHC (systolic, diastolic, and mean PAP) or sociodemographic variables (age and sex), no differences were found (Table 3).

**Table 3:** Comparison of demographic and pulmonary artery pressure variables in the binomial distribution of patients according to pulmonary artery occlusion pressure

Variable	PAOP < 15mmHg	PAOP > 15mmHg	p value
Sex			0.808
Male	56 (27.05)	30 (33.96)	
Female	151 (72.9)	61 (67.03)	
Age (years)	49.74 ± 16.92	53.86 ± 16.22	0.8696
PASP (mmHg)	86.64 ± 27.07	94.23 ± 24.71	0.3755
PADP (mmHg)	34.37 ± 14.81	36.91 ± 15.90	0.4901
PAMP (mmHg)	51.84 ± 17.64	56.24 ± 17.41	0.3994
PAOP (mmHg)	10.68 ± 3.16	22.12 ± 6.58	0.0001

PAOP - pulmonary artery occlusion pressure; PASP - pulmonary artery systolic pressure; PADP - pulmonary artery diastolic pressure; PAMP - pulmonary artery mean pressure. Values are presented as n (%) or mean ± standard deviation.

The presence of potential discrepancies between patients with functional classes I-II and III-IV was evaluated, revealing statistically significant differences in the qualitative variables of mortality (p = 0.040), PH group (p = 0.004), 6MWT (p < 0.001) and final stratification risk (p < 0.001). Additionally, statistically significant differences (p < 0.05) were identified when age, weight, and height and the RHC measurements for systolic, diastolic, mean, pulmonary vascular resistance (PVR), and cardiac output were compared. A difference in TTE measurements was identified only for the TAPSE (p = 0.044). For the 6MWT, differences in the maximum heart rate (p = 0.005), distance in meters (p < 0.001), and, ultimately, the proBNP concentration (p = 0.024) were identified.

**Figure 5:**
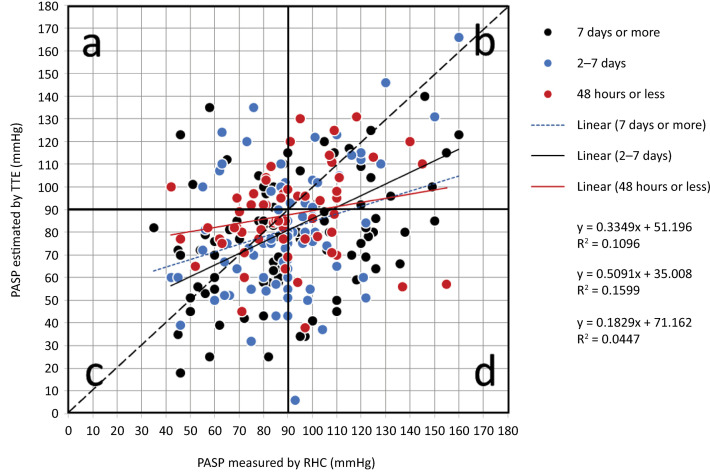
Scatter plot of pulmonary pressures measured via a pulmonary artery catheter and estimations based on transthoracic echocardiography. Scatter plot with the regression line for systolic blood pressure (mmHg) measured by right heart catheterization or estimated by transthoracic echocardiography in all patients. The colors represent the groups of patients defined according to the time difference (in days) between the two measurement techniques in each case, right heart catheterization and transthoracic echocardiography, with intervals of less than 2 days, 2–7 days or more than 7 days. The X-axis represents the gold standard of measurement expressed as the pressure measured during pulmonary artery catheterization. The Y axis represents the values estimated via transthoracic echocardiography. Both axes of the graph have the same value scale. The dashed diagonal represents the line of identity and makes it possible to show which values are identical between catheterization and transthoracic echocardiography. The values that are outside the identity line indicate that the agreement is less than 1. Those nonidentical values are distributed in four quadrants. Quadrant a represents the proportion of values that have been overestimated by transthoracic echocardiography. The values in quadrants b and c represent the high and low values, respectively, of both measurement methods that are not necessarily identical. Quadrant d shows pressure values that have been underestimated by transthoracic echocardiography. PAMP - pulmonary artery mean pressure; RHC - right heart catheterization.

**Figure 6:**
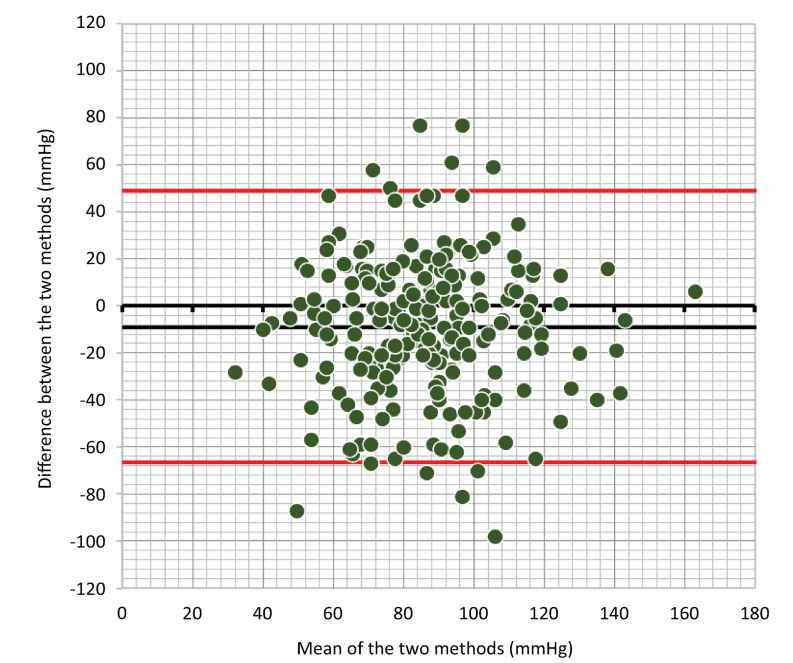
Bland–Altman plot for systolic blood pressure (mmHg) measured by right heart catheterization and transthoracic echocardiography. The red lines correspond to the confidence intervals for the difference vs. the mean of the pulmonary artery pressure variables measured by right heart catheterization and estimated via transthoracic echocardiography.

**Figure 7:**
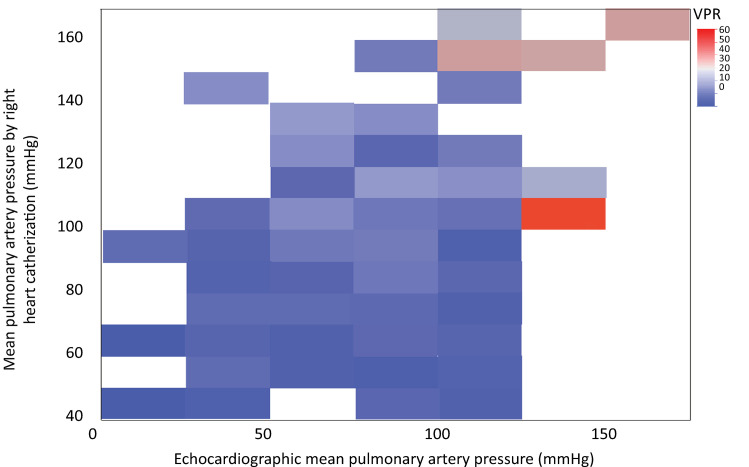
Heat plot for systolic pulmonary pressure measured by ultrasound and right heart catheterization, classified according to pulmonary vascular resistance. Heat gradient of transthoracic echocardiography values plotted on the Y axis and right heart catheterization pressures plotted on the X axis, in which the cold colors (blue) represent low values and the warm colors (red) represent high values. VPR - vascular pulmonary resistance.

**Figure 8:**
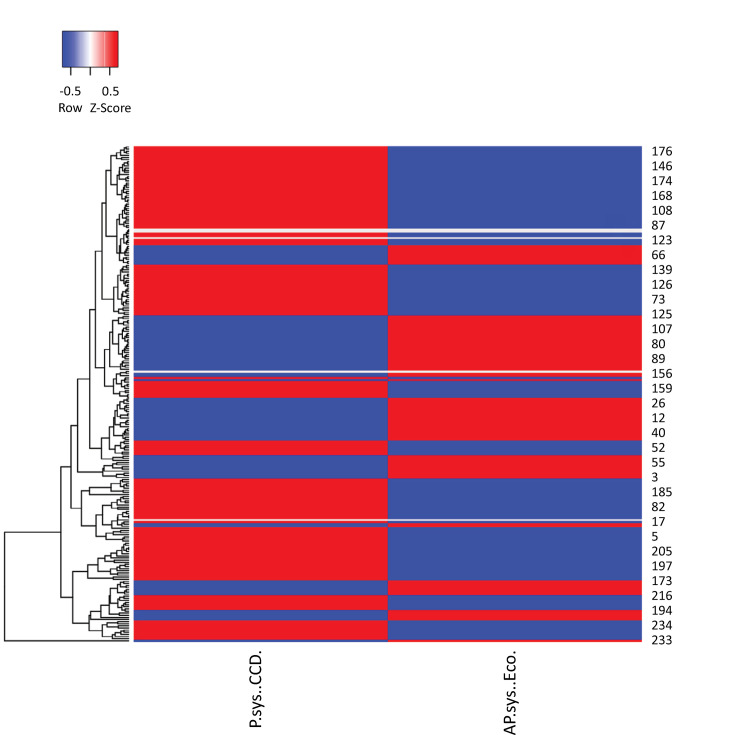
Heat plot for pulmonary artery systolic pressure estimated by transthoracic echocardiography and measured by right heart catheterization. The left sidebar corresponds to the dendrogram generated via an average clustering method with a Euclidean distance measure. PASP - pulmonary artery systolic pressure; RHC - right heart catheterization; TTE - transthoracic echocardiography.

Correlation analysis between the PASP measured by both RHC and TTE revealed statistical significance (mean difference, 8.37 ± 0.22; p < 0.001), with a Spearman’s rho of 0.299 and a Lin correlation coefficient of 0.34 (95%CI 0.23 - 0.44). Given the high variability in the time interval between TTE and RHC, a subgroup analysis was performed of patients who had a time gap between the two examinations of ≤ 7 days (n = 147) and ≤ 48 hours (n = 60). In the first subgroup (≤ 7 days), we obtained a Spearman’s rho of 0.32 (p = 0.0001) and a Lin coefficient of 0.32 (95%CI 0.17 - 0.46), whereas in the second group (≤ 48 hours), we obtained a Spearman’s rho of 0.264 (p = 0.041) and a Lin coefficient of 0.21 [95%CI (> 0.002 - 0.3991)] (Figure 5). Following this low correlation, a concordance analysis was performed to compare the two measurements in the cohort (Figure 6), where a difference between the two methods of 10.14 units (SD 28.19) was detected, establishing 95% confidence limits of -45.11 to 65.39. Similarly, a heat plot was made for the PASP evaluated by the two methods and categorized by PVR. The relationship between the values was determined by employing the heat gradient (changes in color) established by the PVR (with low values represented by cold colors [blue] and high values represented by warm colors [red]) (Figure 7). Additionally, a heat plot was generated to compare the values obtained via RHC and echo, revealing an average clustering method and a Euclidean distance measure (Figure 8), in which it is evident how the colors in the clusters differ between the two methods (TTE and RHC), demonstrating how PASP via TTE is greater or lower [in terms of the number of standard deviations that differ from the mean (z score) for standardized values] than this parameter measured via RHC.

## DISCUSSION

This is the first multicenter study in Colombia to evaluate the correlations and concordance of PASP values estimated from TTE and direct data obtained from invasive measurements with RHC. The scope offered by these results allows us to identify strengths and opportunities for improvement at a multi-institutional level in our country that can improve the care pathway and follow-up in decision-making for patients with PAH and CTEPH. Our data highlights five fundamental concepts.

First, there are difficulties in generalizing findings from previous studies that have evaluated the concordance of pulmonary pressure estimates with RHC. The latter continues to be considered the gold standard for diagnosing PAH or CTEPH. However, there is still debate regarding TTE use or its role in the hemodynamic follow-up of patients with PAH.^(22,23)^Transthoracic echocardiography has proven to be helpful in monitoring right ventricular morphology and function parameters but not in hemodynamic monitoring, which is defined as pulmonary pressure in PAH patients. Previous results contradict the concordance between these two methods in measuring pulmonary pressure during diagnosis.^(24)^In addition, we often need clarification regarding the limited scope of echocardiographic measurements that preclude the estimation of diastolic and mPAP. On the other hand, evaluating the association between pressure parameters estimated by TTE and RHC refers to concordance rather than a linear correlation. Concordance makes it possible to establish to what extent the values are equivalent or, at least, have a correction factor (under or over) that allows simple and clinically valuable assumptions to be made. Our study evaluated intraindividual correlation and concordance but could not define whether these differences were consistent interindividually. If so, the correction factor in the data obtained by the two techniques could be helpful in routine clinical practice.

Second, our study revealed a correlation between the PASP values obtained via TTE and RHC. However, despite its statistical significance in a linear model, the rho value (0.299) demonstrates a weak positive correlation magnitude (defined as ≤ 0.5) and represents a small effect according to Cohen’s criteria,^(25)^which implies a low correlation coefficient. Lin’s correlation index shows a low correlation, with a difference of 10.14 units (SD 28.19) between the two methods. With the above, it is possible to infer that, despite the statistical significance, the correlation between these two parameters for the Colombian population differs from the correlations described in the literature worldwide.^(22,23,26)^ However, a meta-analysis of nine prospective cohorts demonstrated an almost perfect correlation of PASP values by the modified Bernoulli formula between RHC and TTE (r = 0.97, p < 0.001),^(26)^data that differ from the findings of the present study. Nevertheless, despite the high correlation, the low specificity (56%) is striking. Additionally, despite being a meta-analysis, the total sample included 482 patients with high heterogeneity in the precision of the measurement (I2 99.1%, p < 0.0001), which suggests that large-scale studies with a larger sample size and standardization of the acquisition are needed to support this conclusion on a mathematical and scientific basis. The above possibly derives from the population, genomic and sociodemographic differences that the Colombian population presents and that, to date, have not been described; this highlights the importance of the creation of our national policies, given that, as previously described, the criteria of the sixth symposium on pulmonary hypertension do not apply to our population.^(27)^

Third, it is important to highlight that the performance of RHC has difficulties inherent to the test, which limits its inclusion in the follow-up for all patients with PAH or CTEPH in all regions of the country, care centers and other Latin American countries.^(28)^This limitation may impose a risk of bias in the adequate stratification of patients evaluated only with clinical or TTE parameters. Therefore, it is imperative to standardize RHC at the time of patient diagnosis, as well as a more robust set of follow-up studies to identify the behavior of RHC in the diagnosis and follow-up of Colombian patients. Additionally, given the scientific evidence showing the crucial role of the right ventricle and its adaptive capacity as a predictor of mortality,^(3,5,11,17)^the role played by the RHC in allowing the measurement of other hemodynamic variables that enable the assessment of right ventricular function is vital.^(29)^

Fourth, investigating the causes of the low concordance of pulmonary pressure values between the two methods is pertinent. Since they cannot be pinpointed with the present study, we can only speculate about them, as they appear to be multifactorial. There is a potential difference that is evaluator-dependent in the technical aspects of Doppler-derived calculations. In fluid dynamics, Bernoulli’s principle states that an increase in the velocity of a fluid co-occurs with a decrease in the static pressure or a reduction in the potential energy of the liquid. The principle is named after Daniel Bernoulli, who published it in his book “Hydrodynamica” in 1738. Bernoulli deduced that the pressure decreases as the flow velocity increases. However, Leonhard Euler derived Bernoulli’s equation in its usual form in 1752. The principle is applicable only to isentropic flow, and if the effects of irreversible processes (such as turbulence) and nonadiabatic methods (e.g., heat radiation) are minor, they can be neglected. Bernoulli’s principle can then be applied to various types of fluid flow, which results in several forms of Bernoulli’s equation. The simple form of Bernoulli’s equation is valid for incompressible flows (e.g., most liquid and gas flows moving at low Mach numbers). More advanced conditions can be applied to compressible flows with higher Mach numbers (see the derivations of Bernoulli’s equation). Bernoulli’s principle can be derived from the direction of the conservation of energy. The above states that, in a steady flow, the sum of all forms of energy in a fluid along a streamline is the same at all points along that streamline; this requires that the sum of the kinetic, potential, and internal energy remain constant. However, the Bernoulli equation assumes that there are no dissipative forces (i.e., no viscous forces), that the flow is continuous and perfectly laminar (no eddies or vortices), and that the pipe is not distensible or elastic. These conditions are clearly not met in the pulmonary artery or right ventricle and may account for the unequal systolic pressure values both intra- and interindividually. Finally, the distensibility and elastic recoil of the pulmonary artery could also represent underestimated cofactors that decrease the agreement of measurements by the two methods.

Fifth, this study revealed that a subgroup of patients met the criteria for combined PH. Therefore, a subanalysis was performed on patients with combined pre- and postcapillary pulmonary hypertension (n = 93), defined as PAPm > 20mmHg, pulmonary wedge pressure greater than 15mmHg, and PVR > 2 wood units.^(13)^No differences were detected in other catheterization or sociodemographic variables, with patients with wedge pressures lower than 15mmHg taken as referents. This new classification of patients, under the ESC/ERS 2022 guidelines,^(12)^highlights the inclusion of a group of patients with pulmonary wedge pressures greater than 15mmHg^(30)^ and in whom adequate diagnosis and follow-up over time are crucial, given the higher mortality associated with this condition.^(31,32)^

The present study allows us to characterize the PAH and CTEPH problems in the country^(32)^ and indirectly assess the difficulties inherent in accessing diagnostic and treatment strategies in patients with PAH.^(28)^The characterization of our patients, as well as data from the Latin American registry of data on patients with PAH (RELAHP II),^(33)^an incident cohort in Brazil,^(34)^the Recopilar registry in Argentina,^(35)^and reports from a care center in Chile,^(36)^show a predominance of patients with advanced functional class (III-IV), with idiopathic PAH being the most prevalent cause in Group 1 patients. As previously described, our registry and the RELAHP II data highlight the importance of RHC in defining advanced hemodynamic status.^(33)^In a complementary manner, the present study allows us to determine that, for the Colombian population, having a functional class III-IV is associated with more significant mortality, lower values in the distance covered in the 6MWT, and greater severity in the risk strata, which is given by patients with a different distribution between functional class groups for PASP, diastolic PAP, PVR, and cardiac output measured by RHC, as well as TAPSE measured by echocardiography, distance in the 6MWT, concentration of proBNP and peak heart rate during the comprehensive cardiopulmonary exercise test (CPET).

The lack of standardization in management is recognized, in addition to the fact that the information corresponds to different centers with different levels of complexity and experience in the management of PAH, which is our reality and means that many patients still need to complete all the stratification variables. One of the limitations of the present study is that it was impossible to obtain information on perfectly simultaneous data for TTE and RHC in all patients; this is because the registry has a real-life component consisting of analyzing the data available at the cutoff time for the examination. Additional missing information can also be retrieved, but the existing data retain the distribution, dispersion, and associations we have described. Despite having data verification, there were patients for whom no report was available at the time of analysis; this determines a risk of variability in the measurements between the different studies evaluated, which should be considered when these data are interpreted. Notably, in the correlation study, only patients whose total data were available for these two variables were considered. In addition, the absence of perfect synchrony between the time of TTE evaluation and the RHC implies that the clinical situation may have changed during this time; however, this is unlikely to explain the lack of concordance, even for differences of ≤ 48 hours.

Importantly, this Colombian reality highlights the relationship in terms of correlation v*ersus* concordance between the pulmonary artery pressure values that can be estimated via TTE and the pulmonary artery pressure values measured invasively with RHC in our health care environment. This is not only a statistical postulate but also an element that can affect the clinical interpretation of the results and decision-making in the context of real life.

It is essential to note the importance of initiatives such as the present network, as well as the future performance of complementary studies in which the two tests are in perfect synchrony, which allows the analysis and description of the behavior of Latin American populations characterized by their high heterogeneity and which would enable the epidemiological and hemodynamic characterization of patients with PAH.^(28,37)^

## CONCLUSION

A high proportion of patients (but not all) diagnosed with pulmonary arterial hypertension, chronic thromboembolic pulmonary hypertension or combined pulmonary hypertension in Colombia have data from close-in-time echocardiographic and invasive hemodynamic studies at the time of diagnosis. The echocardiographic systolic pulmonary arterial pressure estimates were correlated with the systolic pulmonary arterial pressure values assessed by pulmonary artery catheterization. However, while the correlation between the two measurements is significant it is weak, and the identity of the values between the two methods is practically absent; some of them show large differences, and consequently, their concordance is low. This was evident even in those patients in whom the tests were performed close in time. These results should be disseminated to emphasize the low intrinsic concordance of systolic pulmonary arterial pressure values obtained from noninvasive and invasive techniques in these patients, to restrict the usefulness of the technique individually for each patient considering the degree of difference in the values, and to promote greater use of tricuspid regurgitation jet velocity and right ventricular morphofunctional variables in follow-up and therapeutic decision-making.
